# Myxofibrosarcoma of the sinus piriformis: case report and literature review

**DOI:** 10.1186/1477-7819-10-245

**Published:** 2012-11-15

**Authors:** Zhu Qiubei, Lin Cheng, Xu Yaping, Lin Shunzhang, Fan Jingping

**Affiliations:** 1 Department of ear, nose and throat, Changzheng Hospital, No 415, Fengyang Road, Shanghai, Huangpu District, 200003, China; 2 Department of ear, nose and throat, The 452nd Hospital of PLA, Gongnongyuan Street, Chengdu, Sichuan Province, China

**Keywords:** Myxofibrosarcoma, Sinus piriformis

## Abstract

Myxofibrosarcoma is a common sarcoma in the extremities of older people, but is rare in the head and neck region. Here, we report the case of a 42-year-old male patient in whom myxofibrosarcoma generated from the sinus piriformis. Histopathologically, the tumor was characterized by spindle cellular proliferation with moderate cellular density in fibromyxoid stroma. Immunohistochemically, the tumor cells showed positive reactivity for vimentin, Ki-67, smooth muscle actin, and CD34, but negative staining for S-100. Based on these results, the tumor was diagnosed as a low-grade myxofibrosarcoma. Resection of the tumor was performed via a transcervical approach. The patient’s postoperative clinical course was uneventful and no local recurrence or distant metastasis has been found so far. The pathology, clinical characteristics, and treatment of myxofibrosarcoma are also reviewed.

## Background

As one of the most common sarcomas, myxofibrosarcoma (MFS) is originally described as a myxoid variant of malignant fibrous histiocytoma (MFH), the unique characteristic of which can be ascribed as its frequent occurrence in the subcutaneous tissues of the extremities of older people. Myxofibrosarcoma rarely occurs in the head and neck regions [1], and only a few corresponding cases in these areas have been reported, with 3% to 10% involvement, including the larynx [2], esophagus [3], sphenoid sinus [4,5], mandible [6], maxillary sinus [7], parotid [8,9], orbit [10,11], and infratemporal space [12]. To our knowledge, only one case has so far been reported regarding MFS generated from the hypopharynx [13].

The objective of this work was to report an additional case of MFS arising in the sinus piriformis. The clinical, radiological, and histopathological characteristics of this tumor were also reviewed. Three years after operation, the postoperative clinical course was uneventful and no evidence of recurrence or metastasis and symptoms in the throat were observed in this patient.

## Case presentation

**Figure 1:**
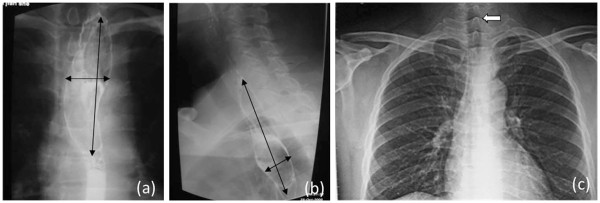
**Barium meal (baM) examination of esophagus showing significant broadening in the cervical segment.** (**a**) Front view (**b**) Side view. (**c**, arrow) X-ray of the chest showed that the trachea leaned to the right.

**Figure 2:**
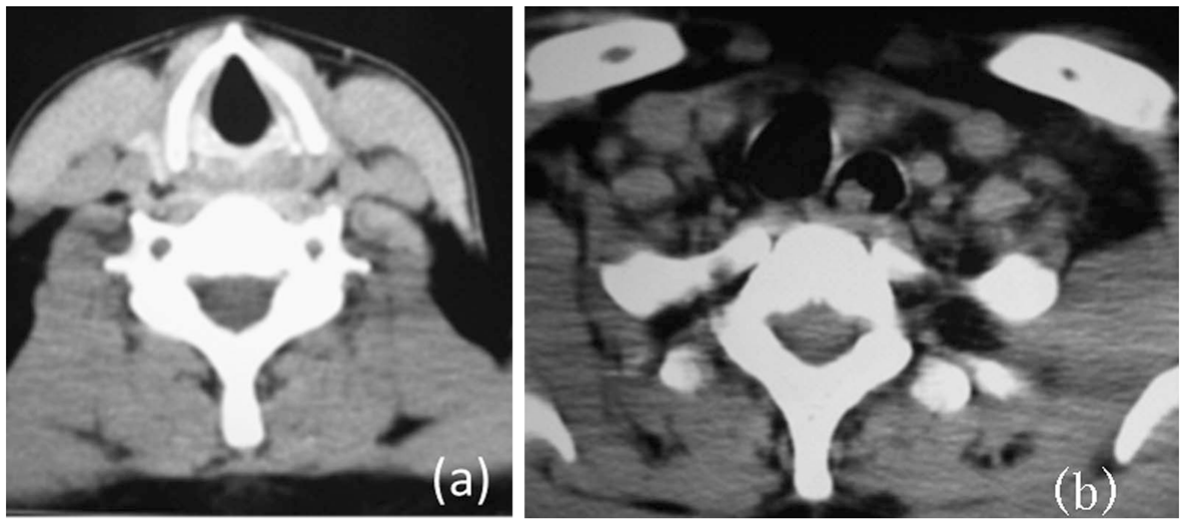
**CT showing the mass in the esophagus.** (**a**) The larynx was normal. (**b**) The esophagus was dilated and the left bronchus was compressed (arrow shows the mass).

**Figure 3:**
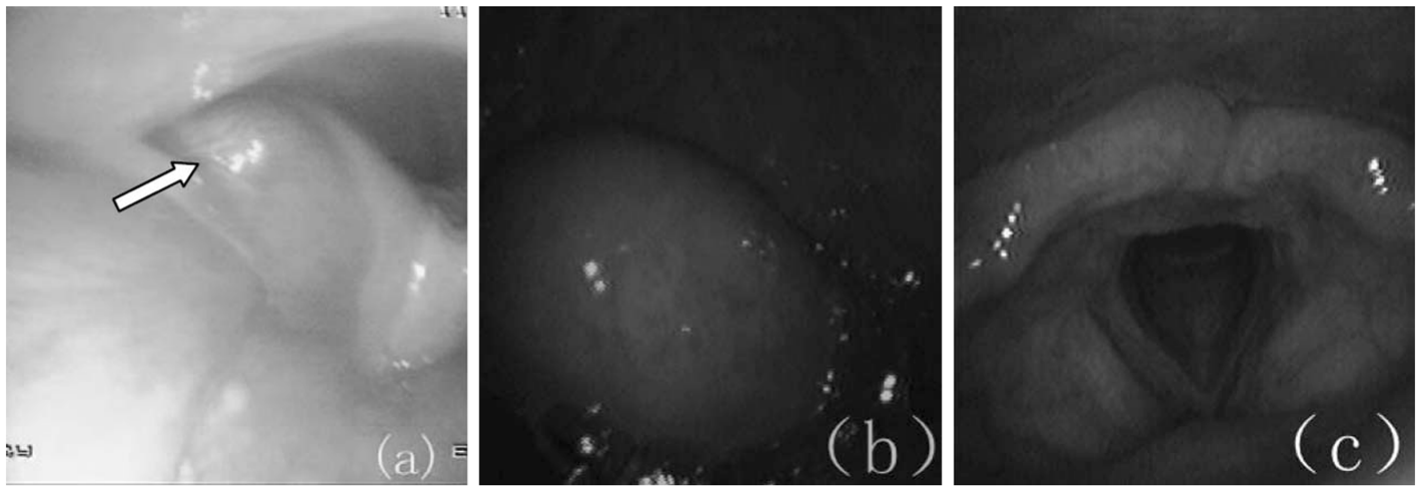
**Local finding of the hypopharynx.** The tumor was located at the hypopharyngeal left sidewall. The surface was smooth and it was the same normal color as the surrounding mucosa. (**a**) Arrow indicates the base of the pedicle, coming from the left piriform sinus. (**b**) Mass in the pharynx when vomiting. (**c**) The larynx was normal.

**Figure 4:**
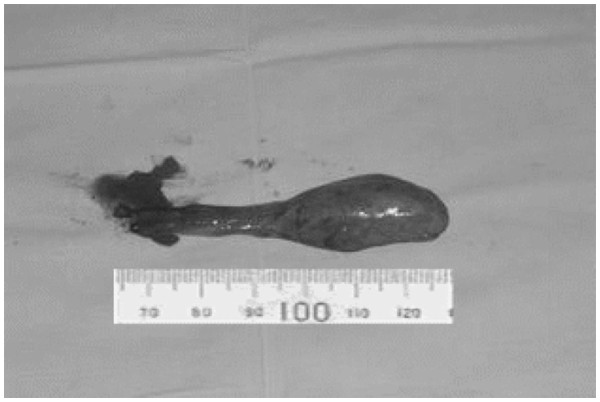
Overall view of the tumor.

A 42-year-old Chinese man had felt an unpleasant sensation in the throat since July 2005. He visited a local otorhinolaryngology clinic and a tumor was found in the hypopharynx. The tumor was resected surgically under a laryngoscope in October 2005. However, the patient still complained of a mass coming from the deep throat sooner after the operation. When vomiting, he could feel the mass coming from his throat into his mouth, and when swallowing, the mass would go downward. A barium meal (baM) examination of the esophagus showed that the cervical segment, about 14 cm from the cutting tooth, was broadened to 50 mm, and the broadened segment was 50 mm long (Figures 1a,b). An X-ray of the chest showed that the trachea leaned to the right (Figure 1c). A computed tomography (CT) scan revealed an irregular-shaped mass in the esophagus, which seemed to originate in the hypopharynx. The mass was 90 mm long and 25 mm wide; the first 40 mm was thin and flat, and the last 50 mm was globular (Figures 2a,b). The patient felt that the mass had been growing slowly over the previous 2 years. Accordingly, he visited our hospital on November 10, 2008. On examination, we found that the surface of the mass was smooth, like normal mucosa. The laryngeal endoscopy and electronic gastroscopy showed that the pedicle of the mass arose from the medial wall of the left sinus piriformis at the level of the aryepiglottic fold (Figures 3a,b,c). The tumor shifted downward and upward when swallowing. There was no relationship between the mass and the esophageal mucosa. The pedicle of the mass was 50 mm long, and the mass itself 40 mm long, and this led to incomplete resection of the mass under the laryngoscope. Based on the histological and the pathological diagnosis of the previous surgery, we supposed that the mass was a recurrent or persistent MFS. We performed tumor resection and wound suture via a transcervical approach on November 28, 2008. The mass was well demarcated, 30 mm × 20 mm in diameter and the cut surface was mucous yellow-gray and translucent (Figure 4).

**Figure 5:**
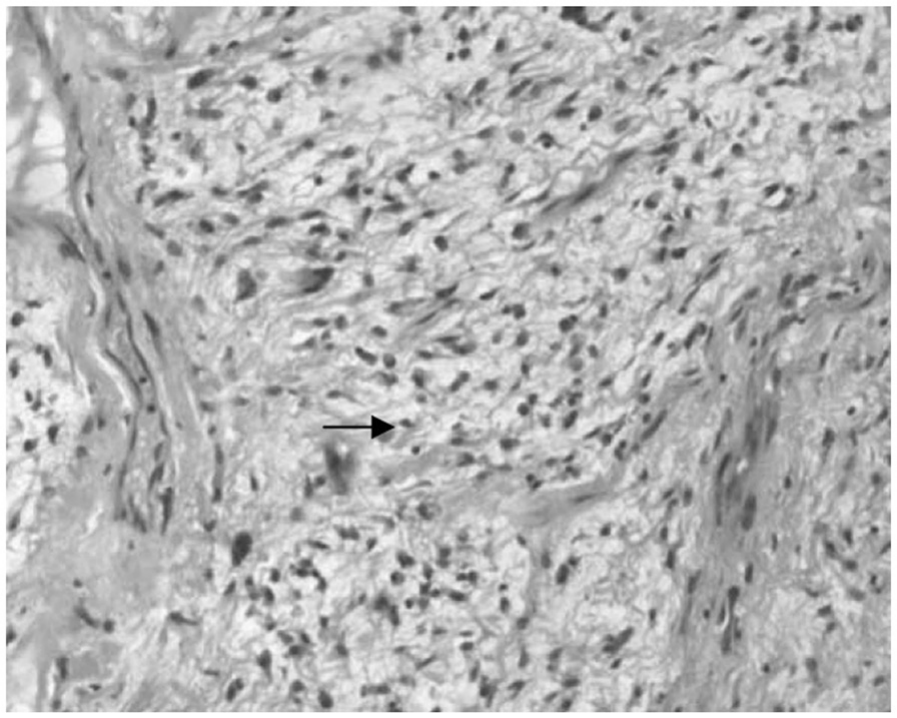
**Spindle cells proliferated in fibrous and myxoid stroma.** Arrow shows tumor cell.

**Figure 6:**
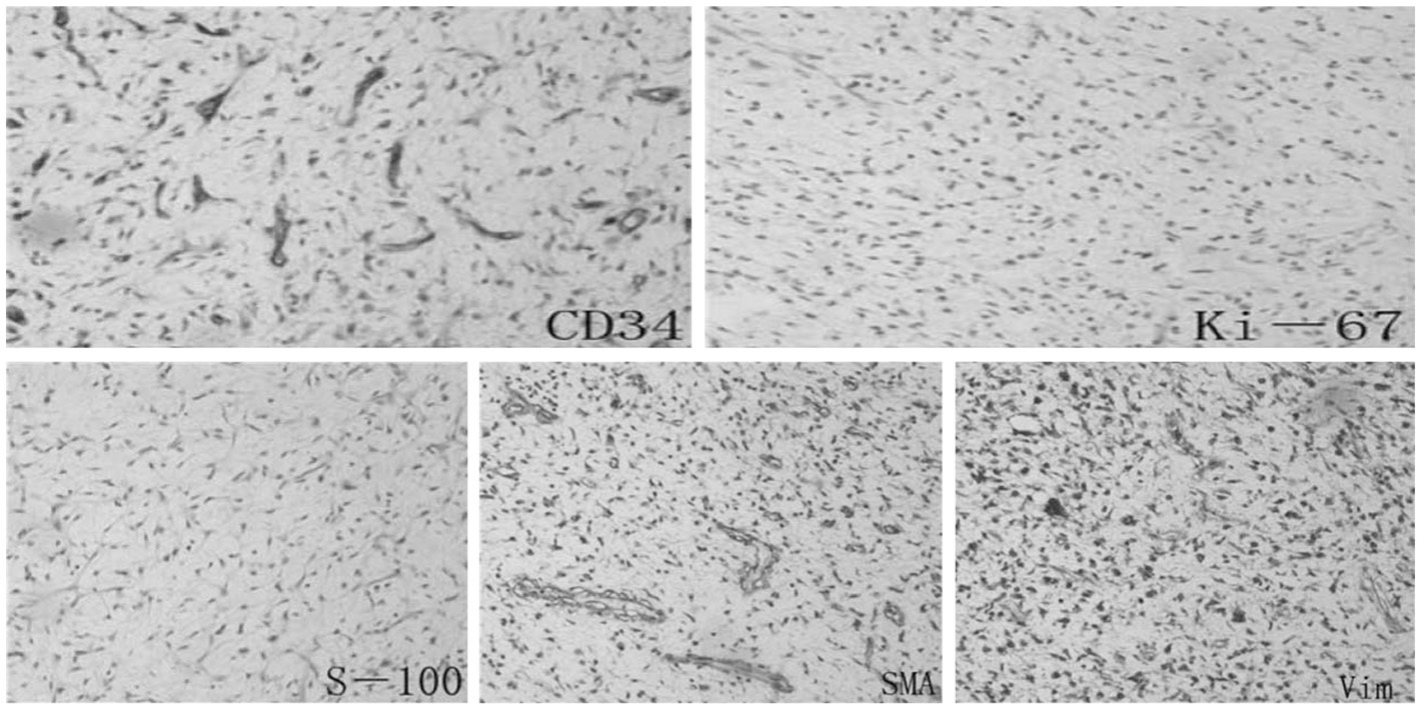
**Immunohistochemical reactivity of the tumor cells.** Positive for vimentin, Ki-67, smooth muscle actin, CD34, but negative for S-100. Vim, vimentin; SMA, smooth muscle actin.

Pathological findings showed typical features of low-grade MFS. The tumor was characterized by spindle-cell proliferation with moderate cellular density in the fibromyxoid stroma (Figure 5). Immunohistochemically, the tumor cells were positive for vimentin, Ki-67, smooth muscle actin (SMA), and CD34, but negative for S-100 (see Figure 6). Thus, the tumor was ultimately diagnosed with low-grade myxofibrosarcoma (myxoid MFH). The surgical margin was sufficient. The postoperative clinical course was uneventful and the patient had no symptoms in the throat after the operation.

## Discussion

Malignant fibrous histiocytoma is the most common malignant soft-tissue tumor in adults [14]. Histopathologically, MFH can be divided into five subtypes: storiform-pleomorphic, myxoid, giant cell, inflammatory, and angiomatoid [15-17]. The term MFS is proposed as a synonym for myxoid MFH and covers a spectrum of malignant fibroblastic lesions, which have cellular distribution, pleomorphism of the nucleus, and mitotic activity that varies from a less cellular lesion with minimal cytologic atypia to a greater cellular lesion with pronounced atypical features [18,19]. Myxofibrosarcoma usually grows slowly and painlessly in the extremities of older people, with a slight male predominance [19]. Superficially located MFS typically consist of multiple variably gelatinous or firmer nodules, with a myxoid cut surface, whereas deep-seated neoplasms often form a single mass with an infiltrative margin. However, MFS is uncommon in the head and neck region. Only 18 cases have been described in the head and neck so far (Table 1), our case being the second in the hypopharynx.

**Table 1:** Cases of myxofibrosarcoma in the head and neck region

**Author**	**Year**	**Sex**	**Age**	**Tumor extent**	**Treatment**	**Results**
Blitzer *et al*. [20]	1981	Male	66	Sphenoid sinus	Radiotherapy	Died after 3 months
Pomerantz *et al*. [21]	1982	Male	58	Maxillary sinus	Surgery	Unknown
Barnes and Kanbour [22]	1988	Female	67	Sphenoid sinus-cavernous sinus	Surgery, adjuvant radiotherapy	Alive after 8 months
Imai *et al*. [11]	2000	Female	52	Orbit	Surgery	Unknown
Lam *et al*. [23]	2002	Male	55	Left sphenoid sinus	Surgery	Alive after 8 months
Iguchi *et al*. [24]	2002	Male	Unknown	Maxillary	Unknown	Unknown
Song and Miller [3]	2002	Male	40	Esophagus	Surgery	Unknown
Nishimura *et al*. [13]	2006	Male	69	Hypopharynx	Surgery	Alive after 16 months
Udaka *et al*. [1]	2006	Male	55	Neck	Surgery	Alive after 27 months
Enoz and Yusufhan [25]	2007	Female	36	Maxillary sinus	Surgery	Alive after 2 years
Gugatschka *et al*. [26]	2010	Male	79	Vocal folds	Surgery	Unknown
Xu *et al*. [8]	2010	Female	37	Parotid	Surgery, radiotherapy	Alive after 8 months
Zhang *et al*. [10]	2010	Female	27	Orbit	Surgery, radiation	Alive after 6 months
Zouloumis *et al*. [6]	2010	Male	23	Mandible	Surgery, radiotherapy	Alive 39 months
Norval *et al*. [7]	2011	Male	69	Maxillary sinus	Radiotherapy, chemotherapy	Died after 1 year
Srinivasan *et al*. [9]	2011	Female	78	Parotid	Surgery, radiotherapy	Died after 24 months
Krishnamurthy *et al*. [12]	2011	Female	42	Infratemporal space	Surgery, radiotherapy	Alive after 26 months
Nakahara *et al*. [27]	2012	Male	52	Maxilla	Surgery, radiotherapy	Alive after 20 months

Histologically, MFS may exhibit various proportions of myxoid matrix with varied cellularity. Thus, it is suggested that MFS be subdivided into four [28] or three [29] grades according to the degree of cellularity, pleomorphism of the nucleus, and mitotic activity. Low-grade MFS shows a hypocellular to moderately cellular architecture with a prominent myxoid matrix. Tumor cells are fusiform, round, or stellate, with ill-defined, slightly eosinophilic cytoplasm and atypical, enlarged, hyperchromatic nuclei (Figure 5). Mitoses can only seldom be seen [1,26]. Most of the tumors have stretched and curved capillaries, and the tumor cells tend to be located along the vessel periphery. Another finding worth mentioning is the presence of prominent elongated, curvilinear, thin-walled blood vessels with a perivascular condensation of tumor cells or inflammatory cells (mainly lymphocytes and plasma cells). Although magnetic resonance imaging and CT scans evidently make a great contribution to the visualization of malignant features, such as local tissue invasion, histopathological examination is recognized as the gold standard, for its capability to provide a definitive diagnosis [30]. Immunohistochemically, low-grade MFS is generally positive for CD-34, vimentin [1], and sometimes for SMA and Ki-67 [31], while negative for S-100 protein. The histopathological findings of the present case were further consistent with a diagnosis of low-grade myxofibrosarcoma and immunoreactivity to vimentin and CD34, probably reflecting the tumor’s primitive fibroblastic nature.

Complete tumor resection with adequate resection margin remains the mainstay for treatment of MFS. The radiotherapy is applied only for recurrent, unresectable lesions or tumors with positive resection margins, to suppress local recurrence and the risk of histologic progression, especially for low-grade MFS. The value of chemotherapy in MFS is still an issue for open debate [5]. Low-grade MFS is considered to have low malignancy, and rarely shows distant metastasis, implying a good short-term prognosis. The overall 5-year survival rate is 60% to 70% [23]. However, the local recurrence rate of the low-grade type is as high (50% to 60%) as that of the high-grade type. It has continuity from low- to high-grade subdivision, showing low-grade areas in high-grade lesions, and a histologic progression of low- to high-grade tumors in recurrences, hence acquiring metastatic potential [15,29]. Therefore, these patients should be placed under careful and long-term follow-up. Complete resection of the tumor was accomplished in our case and this patient showed no recurrence and metastasis three years after the operation.

## Conclusions

In summary, we report a rare case of MFS that generated from the sinus piriformis. Histopathological examination is the gold standard for offering a definitive diagnosis, and the prognosis is accurate after complete resection and careful surveillance. Local recurrence may occur generally with progression of the tumor stage and risk of later metastasis. In this case, the hypothesis that the tumor is recurrent may be reasonable because of the insufficient resection in the previous operation. This emphasizes the recurrent character of MFS and the importance of sufficient resection.

## Consent

Written informed consent was obtained from the patient for publication of this case report and any accompanying images. A copy of the written consent is available for review by the editor-in-chief of this journal.

## Abbreviations

CT: computed tomography; MFH: malignant fibrous histiocytoma; MFS: myxofibrosarcoma; SMA: smooth muscle actin.

## Competing interests

The authors declare that they have no competing interests.

## Authors' contributions

ZQ and LC participated in the design of this study. XY carried out the study, together with LS, who collected important background information and drafted the manuscript. FJ conceived this study, participated in the design, and helped to draft the manuscript. All authors read and approved the final manuscript.

## Authors' information

Zhu Qiubei and Lin Cheng should be regarded as co-first authors.
